# Comparison of Diagnostic Accuracy of Thyroid Cancer With Ultrasound-Guided Fine-Needle Aspiration and Core-Needle Biopsy: A Systematic Review and Meta-Analysis

**DOI:** 10.3389/fendo.2020.00044

**Published:** 2020-02-13

**Authors:** Ling Lan, Yong Luo, Meicen Zhou, Lili Huo, Hailing Chen, Qingyao Zuo, Wei Deng

**Affiliations:** ^1^Endocrinology Department, Beijing Jishuitan Hospital, The Fourth Clinical College of Peking University, Beijing, China; ^2^Department of Urology, Beijing Anzhen Hospital, Capital Medical University, Beijing, China

**Keywords:** biopsy, fine-needle aspiration, meta-analysis, thyroid cancer, systematic review

## Abstract

This systematic review and meta-analysis aimed to evaluate the accuracy of fine-needle aspiration (FNA) and core-needle biopsy (CNB) in diagnosing thyroid cancer. The PubMed, Embase, and Cochrane Library databases were retrieved up to May 2019, and the overall accuracy of FNA and CNB in diagnosing thyroid cancer was evaluated by meta-analysis. The sensitivity, specificity, positive likelihood ratio (PLR), and negative likelihood ratio (NLR) were calculated. The summary receiver operating characteristic (ROC) curve was estimated, and the area under the ROC curve (AUC) was calculated. Ten eligible studies, involving 10,078 patients with 10,842 thyroid nodules, were included. The overall sensitivity and specificity of FNA and CNB for thyroid cancer were 0.72 [95 % confidence interval (CI): 0.69–0.74], 0.99 (95% CI: 0.98–0.99), and 0.83 (95% CI: 0.81–0.85), 0.99 (95% CI: 0.98–0.99), respectively. Other parameters used to assess efficacy included PLR 41.71 (2.15–808.27) and 51.56 (3.20–841.47), NLR 0.31 (0.22–0.42) and 0.22 (0.15–0.32), for FNA and CNB, respectively. Overall, the pooled summary ROC (AUC) value of FNA and CNB was 0.9025 and 0.7926, respectively. No significant difference was observed between the two AUCs of FNA and CNB (*P* = 0.164). FNA and CNB are still similar as first-line diagnostic tools. FNA remains a good first-line method for detecting thyroid malignancies.

## Introduction

Thyroid nodules are common, and more than 95% are benign. However, the National Cancer Institute indicates that thyroid cancer remains the most common endocrine-related cancer. In 2016, 64,330 new cases were reported in the United States. Thyroid cancer accounts for about 3.8% of all new cancer cases (1). Different types of thyroid malignancies have different survival rates, but most thyroid cancers are highly treatable and can be cured on time. This makes an early and accurate diagnosis of malignant nodules critical, as they require immediate surgical resection and adjuvant radioactive iodine treatment (2).

Fine-needle aspiration (FNA) cytology is the primary tool for evaluating thyroid nodules (3). However, the non-negligible part of the thyroid lesion that receives FNA is interpreted as an indeterminate thyroid nodule, representing the gray area of cytology (4). These thyroid cytology samples show that a single cell population with or without colloids does not allow for the identification of malignant and benign lesions (4). In these cases, the International Guidelines (5) recommend the evaluation of almost all patients by diagnostic surgery. In the histological analysis of surgical specimens, a vast majority (70–80%) of thyroid nodules with indeterminate FNA results are benign (6). Therefore, identifying parameters that may serve as potential markers of malignancy has a significant clinical value. Some of the molecular, cytological, ultrasound, and clinical studies investigated these parameters but yielded controversial results (7–9). Some recent studies reported that the microscopic histological examination of the core-needle biopsy (CNB) could diagnose a large number of uncertain thyroid nodules. This technique has been widely used worldwide (10). It is particularly important in clinical practice that diagnostic surgery can avoid thyroid nodules that are considered benign CNB. Also, the incidence of minor complications of CNB is very low, which is beneficial for patients (11).

Several recent studies showed that CNB could effectively reduce the non-diagnostic rate of thyroid nodules (12–14), and unnecessary and/or diagnostic surgery (15), initially showing non-diagnostic or uncertain results through FNA (16). A recent small-group pilot study (31 patients) reported that the first-line use of CNB was more effective in suspected thyroid nodules compared with FNA (17). Although CNB has an advantage over thyroid nodules with previously non-diagnostic or indeterminate results, the small sample size may lead to inconclusive results on using CNB in evaluating thyroid nodules. This systematic review and meta-analysis compared the accuracy of CNB and FNA in diagnosing thyroid cancer.

## Materials and Methods

This meta-analysis was based on the Preferred Reporting Items for Systematic Reviews and Meta-analysis guidelines (18).

### Search Strategy

As of May 1, 2019, a comprehensive literature search was conducted on electronic databases PubMed (www.pubmed.gov), Embase (www.embase.com), and Cochrane Library (www.cochranelibrary.com) using the following search terms and combinations: “fine needle aspiration or FNA,” “core needle biopsy or CNB,” “thyroid nodules,” and “malignancy.” Also, published research and review studies were manually searched. No language restrictions are applied.

### Inclusion and Exclusion Criteria

The inclusion criteria were as follows: (1) prospective or retrospective studies involving patients with thyroid nodules using FNA and CNB evaluations; (2) surgical histology as the gold standard; (3) data extracted to construct at least a 2 × 2 test performance table. The exclusion criteria were as follows: investigations of cell lines or animals; reviews, case reports, letters, or meeting records; and insufficient data. Additionally, if more than one study was published using the same case series, the study with the largest sample size was selected.

### Data Extraction and Quality Assessment

Based on the aforementioned criteria, the two authors carefully extracted information from all eligible studies. Data of general research characteristics were extracted from each study: first author's surname, year of publication, country, gender, mean age, number of patients, number of nodules, period of enrollment, study type, diagnostic methods, and number of true positives, true negatives, false positives, and false negatives. The differences were resolved through discussion and consensus. Two reviewers independently assessed the eligibility of each study based on the aforementioned inclusion criteria and assessed the quality of the methodology based on the Diagnostic Accuracy Research Quality Assessment-2 (QUADAS-2) tool (19). The risk of bias was rated as “low,” “high,” or “unclear,” corresponding to a score of “1,” “0,” and “0,” respectively. The study awarded a cumulative score higher than or equal to 6 was considered as high quality.

### Statistic Analysis

Meta DISC1.4 (XI Cochrane Symposium, Barcelona, Spain) was used for data analysis. The pooled sensitivity, specificity, and area under the curve (AUC) were calculated to assess the diagnostic value of FNA and CNB. A heterogeneity analysis was performed using the Cochran Q test and the Higgins *I* square test. If *P* < 0.1 or *I*^2^ > 50%, a random-effects model was applied. If *P* > 0.1 or *I*^2^ <50%, a fixed-effects model was used. Finally, a summary receiver operating characteristic (SROC) curve was drawn based on the literature, and the area under the receiver operating characteristic curve (SAUC) was calculated. The relative impact of each study on the overall evaluation was assessed by deleting a study for sensitivity analysis. In addition, subgroup analysis and meta-regression were employed to trace the potential sources of study heterogeneity. Publication bias was evaluated by the visual inspection of the symmetry of the funnel plot and the assessment of Begg's and Egger's tests. The trim-and fill-analysis was applied in the case of publication bias. All results showed a 95% confidence interval (CI), and two-sided *P* < 0.05 was considered statistically significant.

## Results

### Characteristics of Including Studies

**Figure 1:**
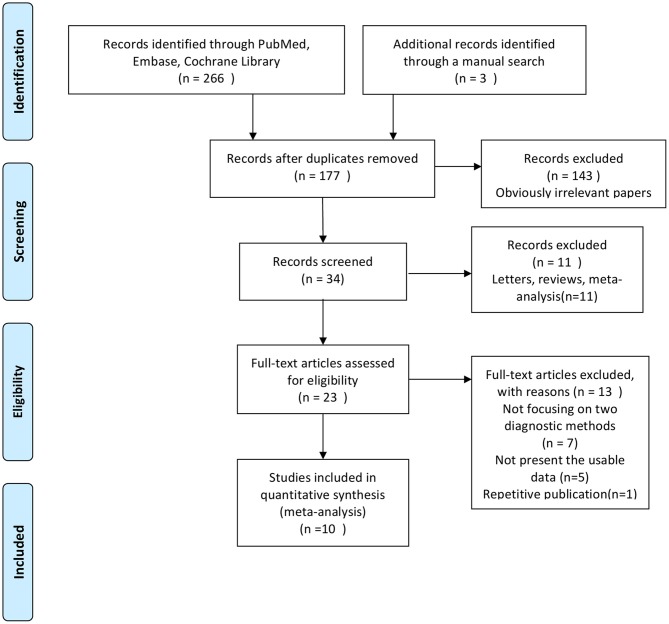
Flow diagram for identification of studies.

**Figure 2:**
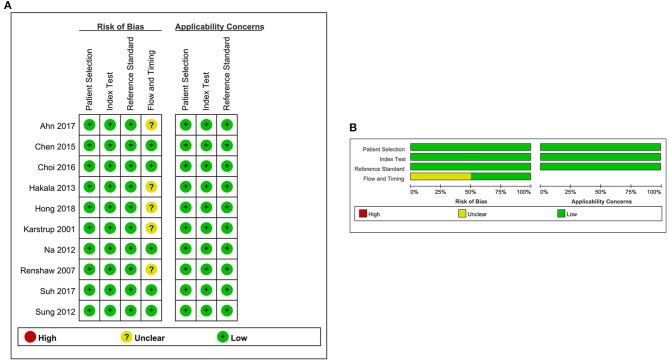
Risk-of-bias and applicability concerns summary for each domain of the QUADAS-2 for each included study. **(A)** Risk-of-bias summary. **(B)** Risk-of-bias graph. Symbols. (+), low risk of bias; (?), unclear risk of bias; (–), high risk of bias.

Figure 1 shows the study selection procedure. A comprehensive search yielded 266 studies. The manual review of the references of retrieved studies on the use of FNA-CNB in thyroid nodules yielded three additional studies that met the inclusion criteria for this analysis. After removing duplicate studies and those containing unspecific data that did not meet the inclusion criteria, 10 studies (12, 14, 20–27) were finally included in the present meta-analysis. Table 1 illustrates the characteristics of all the studies included in this meta-analysis. These studies were published between 2001 and 2018 and conducted in four countries (the USA, Denmark, Korea, and Finland). The total number of enrolled patients was 10,078, with individual samples ranging from 52 to 4,553. The reported mean age of the patients ranged from 4.5 to 95.3 years across the eligible studies. The quality of the included studies was assessed using QUADAS-2, as shown in Figure 2. All included studies were of high quality.

**Table 1:** Characteristics of the studies included in this meta-analysis.

**References**	**Country**	**Male (%)**	**Mean age**	**Patients(N)**	**No. of nodules**	**Period of enrollment**	**Study type**	**Diagnostic methods**
Karstrup et al. (20)	Denmark	17	51 (33–81)	77	77	1997–1999	Retrospective	FNA, CNB
Renshaw et al. (21)	USA	20	52 (14–86)	377	377	2000–2006	Retrospective	FNA, CNB
Na et al. (12)	Korea	13	46 ± 11.7	220	225	2009–2010	Prospective	FNA, CNB
Sung et al. (14)	Korea	16	44.32 ± 11.86	538	555	2008–2009	Retrospective	FNA, CNB
Hakala et al. (23)	Finland	23	53 ± 17	52	52	2010–2011	Prospective	FNA, CNB
Chen et al. (21)	USA	21	58 (14–88)	350	461	2007–2011	Retrospective	FNA, CNB
Choi et al. (22)	Korea	21	55.5 (25–81)	505	505	2008–2013	Retrospective	FNA, CNB
Ahn et al. (20)	Korea	22	50.7 (4.5–95.3)	2,187	2,406	2004–2014	Retrospective	FNA, CNB
Suh et al. (27)	Korea	21	53.4 ± 12.6	4,553	4,822	2013	Retrospective	FNA, CNB
Hong et al. (24)	Korea	18	46.9 ± 12.9	1,219	1,362	2010–2014	Retrospective	FNA, CNB

*FNA, fine-needle aspiration; CNB, core needle biopsy*.

### Quantitative Synthesis

**Figure 3:**
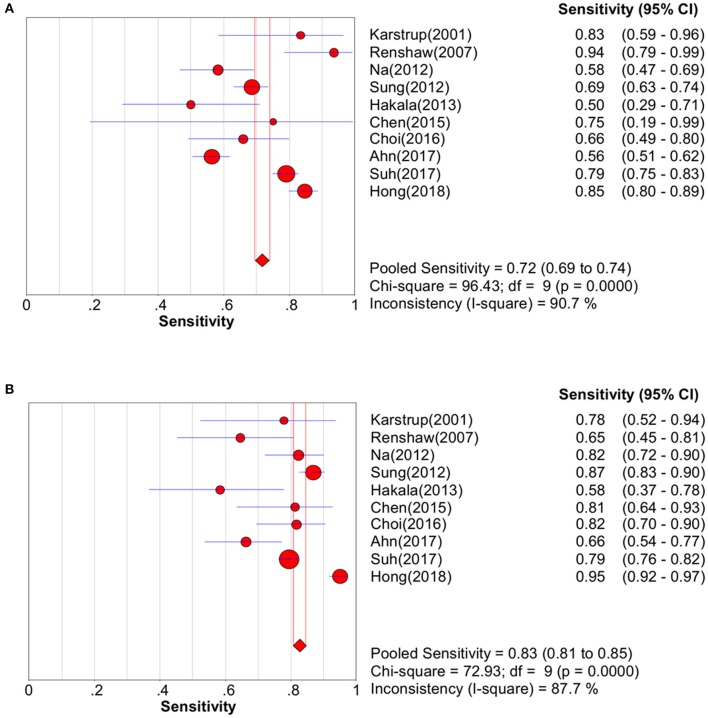
Pooled sensitivity forest plot of two methods in diagnosing thyroid cancer. **(A)** FNA; **(B)** CNB.

**Figure 4:**
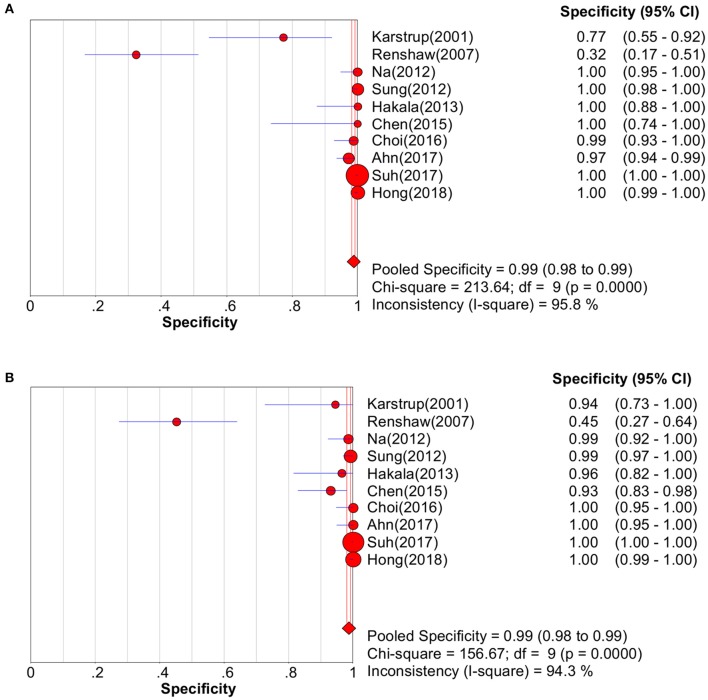
Pooled specificity forest plot of two methods in diagnosing thyroid cancer. **(A)** FNA; **(B)** CNB.

**Figure 5:**
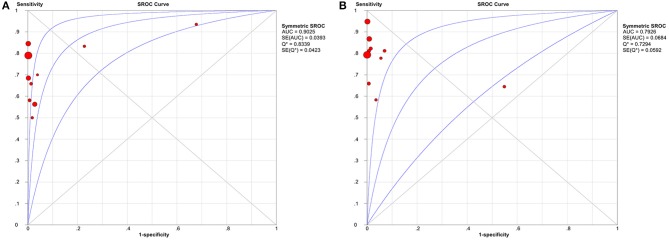
Summary receiver operator characteristic curve (SROC) with the area under the ROC curve (AUC) of the two methods in diagnosing thyroid cancer. **(A)** FNA; **(B)** CNB. AUC, area under the ROC curve; SROC, summary receiver operator characteristic curve.

Study data and individual diagnostic estimates are summarized in Table 2. Compared with the gold standard, the random-effects model meta-analysis of the 11 studies showed a pooled sensitivity of 0.72 (95% CI: 0.69–0.74) (Figure 3A) and a pooled specificity of 0.99 (95% CI: 0.98–0.99) (Figure 4A) for diagnosing benign and malignant lesions of thyroid nodules with FNA. For CNB, the pooled sensitivity and specificity were 0.83 (95% CI: 0.81–0.85) (Figure 3B) and 0.99 (95% CI: 0.98–0.99) (Figure 4B), respectively. The pooled positive likelihood ratio of FNA and CNB was 41.71 (2.15–808.27) and 51.56 (3.20–841.47), respectively; the pooled negative likelihood ratio for FNA and CNB was 0.31 (0.22–0.42) and 0.22 (0.15–0.32), respectively, (Table 2). The pooled SROC (AUC) value of FNA and CNB was 0.9025 and 0.7926, respectively; the pooled SE (AUC) value of FNA and CNB was 0.0393 and 0.0684, respectively (Figure 5). No significant difference was observed between the two AUCs of FNA and CNB (*P* = 0.164). The sensitivity analysis evaluated the impact of a single data set on the summary results by deleting each eligible study in turn. The overall results remained the same after removing any single study (Table 3).

**Table 2:** Summary of results of the studies included in this meta-analysis.

**References**	**Diagnostic methods**	**TP**	**FP**	**FN**	**TN**	**Sensitivity (%)**	**Specificity (%)**	**PLR**	**NLR**
Karstrup et al. (25)	FNA	15	5	3	17	0.83 (0.59, 0.96)	0.77 (0.55, 0.92)	3.67 (1.65, 8.14)	0.22 (0.07, 0.62)
	CNB	14	1	4	17	0.78 (0.52, 0.94)	0.94 (0.73, 1.00)	14.00 (2.05, 95.56)	0.24 (0.10, 0.56)
Renshaw and Pinnar (26)	FNA	29	21	2	10	0.94 (0.79, 0.99)	0.32 (0.17, 0.51)	1.38 (1.06, 1.79)	0.20 (0.05, 0.84)
	CNB	20	17	11	14	0.65 (0.45, 0.81)	0.45 (0.27, 0.64)	1.18 (0.78, 1.78)	0.79 (0.43, 1.45)
Na et al. (12)	FNA	46	0	33	70	0.58 (0.47, 0.69)	1.00 (0.95, 1.00)	82.54 (5.18, 1314.94)	0.42 (0.33, 0.55)
	CNB	65	1	14	69	0.82 (0.72, 0.90)	0.99 (0.92, 1.00)	57.59 (8.21, 404.25)	0.18 (0.11, 0.29)
Sung et al. (14)	FNA	218	0	100	237	0.69 (0.63, 0.74)	1.00 (0.98, 1.00)	326.04 (20.43, 5202.61)	0.32 (0.27, 0.37)
	CNB	276	2	42	235	0.87 (0.83, 0.90)	0.99 (0.97, 1.00)	102.85 (25.86, 409.11)	0.13 (0.10, 0.18)
Hakala et al. (23)	FNA	12	0	12	28	0.50 (0.29, 0.71)	1.00 (0.88, 1.00)	29 (1.81, 465.42)	0.51 (0.34, 0.76)
	CNB	14	1	10	27	0.58 (0.37, 0.78)	0.96 (0.82, 1.00)	16.33 (2.31, 115.28)	0.43 (0.27, 0.70)
Chen et al. (21)	FNA	3	0	1	12	0.75 (0.19, 0.99)	1.00 (0.74, 1.00)	18.2 (1.13, 292.75)	0.31 (0.08, 1.20)
	CNB	26	4	6	53	0.81 (0.64, 0.93)	0.93 (0.83, 0.98)	11.58 (4.44, 30.22)	0.20 (0.10, 0.42)
Choi et al. (22)	FNA	27	1	14	75	0.66 (0.49, 0.80)	0.99 (0.93, 1.00)	50.05 (7.05, 355.12)	0.35 (0.23, 0.53)
	CNB	49	0	11	69	0.82 (0.70, 0.90)	1.00 (0.95, 1.00)	113.61 (7.16, 1803.15)	0.19 (0.11, 0.32)
Ahn et al. (20)	FNA	174	5	135	176	0.56 (0.51, 0.62)	0.97 (0.94, 0.99)	20.38 (8.54, 48.65)	0.45 (0.39, 0.51)
	CNB	45	0	23	71	0.66 (0.54, 0.77)	1.00 (0.95, 1.00)	94.96 (5.97, 1511.4)	0.34 (0.25, 0.48)
Suh et al. (27)	FNA	340	3	90	1,757	0.79 (0.75, 0.83)	1.00 (1.00, 1.00)	463.88 (149.6, 1438.4)	0.21 (0.17, 0.25)
	CNB	524	0	136	1,043	0.79 (0.76, 0.82)	1.00 (1.00, 1.00)	1656.82 (103.67, 26478.25)	0.21 (0.18, 0.24)
Hong et al. (24)	FNA	238	0	43	405	0.85 (0.80, 0.89)	1.00 (0.99, 1.00)	686.74 (43.01, 10965.62)	0.15 (0.12, 0.20)
	CNB	267	0	14	405	0.95 (0.92, 0.97)	1.00 (0.99, 1.00)	770.25 (48.25, 12295.09)	0.05 (0.03, 0.08)
Pooled value	FNA	1,409	36	451	3,031	0.72 (0.69, 0.74)	0.99 (0.98, 0.99)	41.71 (2.15, 808.27)	0.31 (0.22, 0.42)
	CNB	1,476	26	293	2,180	0.83 (0.81, 0.85)	0.99 (0.98, 0.99)	51.56 (3.20, 841.47)	0.22 (0.15, 0.32)

*FNA, fine-needle aspiration; CNB, core needle biopsy; FN, False-negative; FP, False-positive; TN, True-negative; TP, True-positive; PLR, positive likelihood ratio; NLR, negative likelihood ratio*.

**Table 3:** The influence of individual studies using the leave-one-out approach.

**Study excluded**	**Diagnostic methods**	**Sensitivity (95% CI)**	**Specificity (95% CI)**	**AUC**
Karstrup et al. (25)	FNA	0.72 (0.69, 0.74)	0.99 (0.98, 0.99)	0.91
	CNB	0.83 (0.81, 0.85)	0.99 (0.98, 0.99)	0.79
Renshaw and Pinnar (26)	FNA	0.71 (0.69, 0.74)	0.99 (0.99, 1.00)	0.83
	CNB	0.83 (0.81, 0.85)	1.00 (0.99, 1.00)	0.88
Na et al. (12)	FNA	0.73 (0.70, 0.75)	0.99 (0.98, 0.99)	0.90
	CNB	0.83 (0.81, 0.85)	0.99 (0.98, 0.99)	0.79
Sung et al. (14)	FNA	0.73 (0.70, 0.75)	0.99 (0.98, 0.99)	0.90
	CNB	0.82 (0.79, 0.84)	0.99 (0.98, 0.99)	0.78
Hakala et al. (23)	FNA	0.72 (0.70, 0.74)	0.99 (0.98, 0.99)	0.91
	CNB	0.83 (0.81, 0.85)	0.99 (0.98, 0.99)	0.79
Chen et al. (21)	FNA	0.72(0.69, 0.74)	0.99 (0.98, 0.99)	0.90
	CNB	0.83 (0.81, 0.85)	0.99 (0.98, 0.99)	0.77
Choi et al. (22)	FNA	0.72 (0.70, 0.74)	0.99 (0.98, 0.99)	0.90
	CNB	0.83 (0.81, 0.85)	0.99 (0.98, 0.99)	0.79
Ahn et al. (20)	FNA	0.76 (0.73, 0.78)	0.99 (0.98, 0.99)	0.92
	CNB	0.83 (0.82, 0.85)	0.99 (0.98, 0.99)	0.79
Suh et al. (27)	FNA	0.69 (0.66, 0.72)	0.97 (0.96, 0.98)	0.89
	CNB	0.85 (0.83, 0.87)	0.97 (0.96, 0.98)	0.78
Hong et al. (24)	FNA	0.69 (0.66, 0.71)	0.99 (0.98, 0.99)	0.90
	CNB	0.80 (0.78, 0.82)	0.98 (0.98, 0.99)	0.79

*FNA, fine-needle aspiration; CNB, core needle biopsy; AUC, the area under the curve*.

#### Subgroup Analyses

Due to the existence of significant heterogeneity across the whole analyses, subgroups were analyzed depending on the country, number of patients, period of enrollment, and study type. As exemplified in Table 4, FNA achieved a high AUC value of 0.90 in the diagnosis of thyroid cancer (overall), especially in pre-2010 enrollment (AUC = 0.91) and in a retrospective study (AUC = 0.91). Moreover, stratified analyses in terms of country evidenced that CNB presented an AUC of 0.97 in Asian countries better than in occidental countries (AUC = 0.77).

**Table 4:** Subgroup analysis of diagnostic effect.

**Subgroup**	**Diagnostic methods**	**Number of studies**	**Sensitivity (95% CI)**	**Specificity (95% CI)**	**AUC**
Overall	FNA	10	0.72 (0.69, 0.74)	0.99 (0.98, 0.99)	0.90
	CNB	10	0.83 (0.81, 0.85)	0.99 (0.98, 0.99)	0.79
**Country**					
Occidental	FNA	4	0.77 (0.66, 0.86)	0.72 (0.62, 0.81)	0.89
	CNB	4	0.70 (0.61, 0.79)	0.83 (0.75, 0.89)	0.77
Asian	FNA	6	0.72 (0.69, 0.74)	1.00 (0.98, 1.00)	0.89
	CNB	6	0.84 (0.82, 0.85)	1.00 (1.00, 1.00)	0.96
**Number of patients**					
≤ 500	FNA	5	0.67 (0.59, 0.75)	0.84 (0.78, 0.89)	0.89
	CNB	5	0.76 (0.69, 0.82)	0.88 (0.83, 0.92)	0.77
>500	FNA	5	0.72 (0.70, 0.75)	1.00 (0.99, 1.00)	0.90
	CNB	5	0.84 (0.82, 0.86)	1.00 (1.00, 1.00)	0.97
**Period of enrollment**					
Pre-2010	FNA	4	0.69 (0.65, 0.73)	0.93 (0.90, 0.95)	0.91
	CNB	4	0.84 (0.80, 0.87)	0.94 (0.91, 0.96)	0.76
Post-2010	FNA	6	0.73 (0.70, 0.76)	1.00 (0.99, 1.00)	0.88
	CNB	6	0.82 (0.80, 0.84)	1.00 (0.99, 1.00)	0.86
**Study type**					
Prospective	FNA	2	0.56 (0.46, 0.66)	1.00 (0.96, 1.00)	——
	CNB	2	0.77 (0.67, 0.84)	0.98 (0.93, 1.00)	——
Retrospective	FNA	8	0.73 (0.71, 0.75)	0.99 (0.98, 0.99)	0.91
	CNB	8	0.83 (0.81, 0.85)	0.99 (0.98, 0.99)	0.80

*FNA, fine-needle aspiration; CNB, core needle biopsy; AUC, the area under the curve*.

#### Heterogeneity Analysis

The source of heterogeneity was examined by a meta-regression analysis. None of the examined factors, including country (FNA: *P* = 0.657; CNB: *P* = 0.187), number of patients (FNA: *P* = 0.523; CNB: *P* = 0.296), period of enrollment (FNA: *P* = 0.771; CNB: *P* = 0.666), and study type (FNA: *P* = 0.333; CNB: *P* = 0.346), was responsible for heterogeneity across studies in meta-regression.

#### Publication Bias

**Figure 6:**
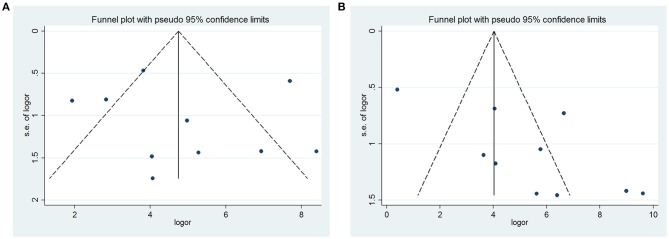
Funnel plot for publication bias test. Each point represents a separate study for the indicated association. **(A)** FNA; **(B)** CNB.

Finally, the Begg's and Egger's regression tests showed no evidence of asymmetrical distribution in the funnel plot in FNA (Begg's test *P* = 0.858; Egger's test *P* = 0.766) (Figure 6A). However, the *P*-value of Begg's test confirmed the existence of publication bias for CNB (Begg's test *P* = 0.210; Egger's test *P* = 0.017; Figure 6B). The trim-and-fill method showed no need for additional studies (Figure S1).

## Discussion

This systematic review and meta-analysis evaluated the accuracy of FNA and CNB in diagnosing thyroid malignancy. The study found a pooled sensitivity of 0.72 (95% CI: 0.69–0.74) and a pooled specificity of 0.99 (95% CI: 0.98–0.99) for FNA. For CNB, the pooled sensitivity and specificity were 0.83 (95% CI: 0.81–0.85) and 0.99 (95% CI: 0.98–0.99), respectively. The pooled SROC (AUC) value of FNA and CNB was 0.9025 and 0.7926, respectively. No significant difference was observed between the two AUCs of FNA and CNB (*P* = 0.164).

Besides pooling the diagnostic characteristics of the index test, identification of heterogeneity is also an important goal of a meta-analysis. The present study assessed the between-study heterogeneity using subgroup and meta-regression analyses. The subgroup analysis indicated that FNA achieved a high AUC value of 0.90 in the diagnosis of thyroid cancer (overall), especially in pre-2010 enrollment (AUC = 0.91) and in a retrospective study (AUC = 0.91). Moreover, stratified analyses in terms of country evidenced that CNB presented an AUC of 0.97 in Asian countries better than in occidental countries (AUC = 0.77). However, none of the examined factors, including country, number of patients, period of enrollment, and study type, was responsible for heterogeneity across studies in meta-regression. These heterogeneities might be due to technical differences in institutions or operators, nodule characteristics, number of passages, or lack of standardized pathological criteria for CNB.

Previous meta-analytical studies yielded conflicting results. Li et al. found no significant differences in the values of the preoperative diagnosis of thyroid nodules between FNA and CNB (28). The two meta-analyses by Tandon et al. (29) and Novoa et al. (30) evaluated the role of FNA and CNB in the diagnosis of head and neck malignancies. Both studies found that CNB was more accurate and specific than FNA, and its negative predictive value was better when applied to all head and neck tumors. However, neither of these studies compared the diagnostic value of FNA and CNB for thyroid malignancies. The present meta-analysis, comprising 10,078 patients with 10,842 thyroid nodules from 10 studies, was the largest study investigating the accuracy of FNA and CNB in diagnosing thyroid malignancy.

In clinical follow-up and surgery for thyroid nodules, FNA is a classic, cost-effective, minimally invasive, easy-to-apply standard of choice. However, the rate of dissatisfaction/non-diagnosis results varies (2–40%) among centers (2, 5, 31, 32). These results are directly related to the technical capabilities of the personnel performing the procedures and evaluating the materials. Besides, among observers, the incidence of AUS/FLUS (a heterogeneous diagnostic group that may lead to uncertainty in patient treatment) varies (3–32.2%) (2, 5, 31, 32). Recent studies explored the potential role of CNB as a first-line tool for diagnosing thyroid nodules. A study showed that CNB had low rates of non-diagnostic results (1.3%), inconclusive results (5.9%), complications (0.2%), high diagnostic accuracy (97.6%), and unnecessary surgery (0.5%) (33). In another study, the diagnostic accuracy of CNB was significantly higher than that of FNA (96.8 vs. 78%, *P* < 0.001), and the incidence of false-negative and uncertain results in suspected US characteristic nodules reduced (17). Hong et al. (24) suggested that CNB could prevent unnecessary repetitive biopsy procedures or diagnostic surgery because 13.5% of nodules had no conclusive results. Also, CNB could achieve additional malignant diagnosis in 27% of malignant tumors compared with FNA. When executed by experienced operators, CNB has been reported as safe (34) and tolerable (11). Therefore, although FNA has been widely used as a first-line diagnostic tool, CNB can be used by experienced operators as an alternative first-line diagnostic tool for thyroid nodules.

This meta-analysis had several limitations. First, it was a retrospective study, and the number of patients in some studies was relatively small. Second, the meta-analysis showed considerable heterogeneity in the pooled proportions. Third, differences existed in the sample collection technology. For example, FNA could be accomplished by capillary or aspiration techniques, and some retrospective studies did not specify which techniques were used.

In summary, FNA and CNB are still similar as first-line diagnostic tools. FNA remains a good first-line method for detecting thyroid malignancies. Additional controlled studies on larger, homogeneous patient groups are needed to validate further the practicability of the aforementioned two methods.

## Author Contributions

LL and YL carried out the studies, participated in collecting data, and drafted the manuscript. LL and MZ performed the statistical analysis and participated in its design. LH, HC, and WD helped to draft the manuscript. All authors read and approved the final manuscript.

### Conflict of Interest

The authors declare that the research was conducted in the absence of any commercial or financial relationships that could be construed as a potential conflict of interest.

## Supplementary Material

Figure S1 Filled funnel plot of any PEP using trim-and-fill method.
