# Supplementary figures and images for: Comparison of Diagnostic Accuracy of Thyroid Cancer With Ultrasound-Guided Fine-Needle Aspiration and Core-Needle Biopsy: A Systematic Review and Meta-Analysis
Source: Front Endocrinol (Lausanne). 2020 Feb 13;11:44. doi: 10.3389/fendo.2020.00044 (PMC7033392; doi:10.3389/fendo.2020.00044)

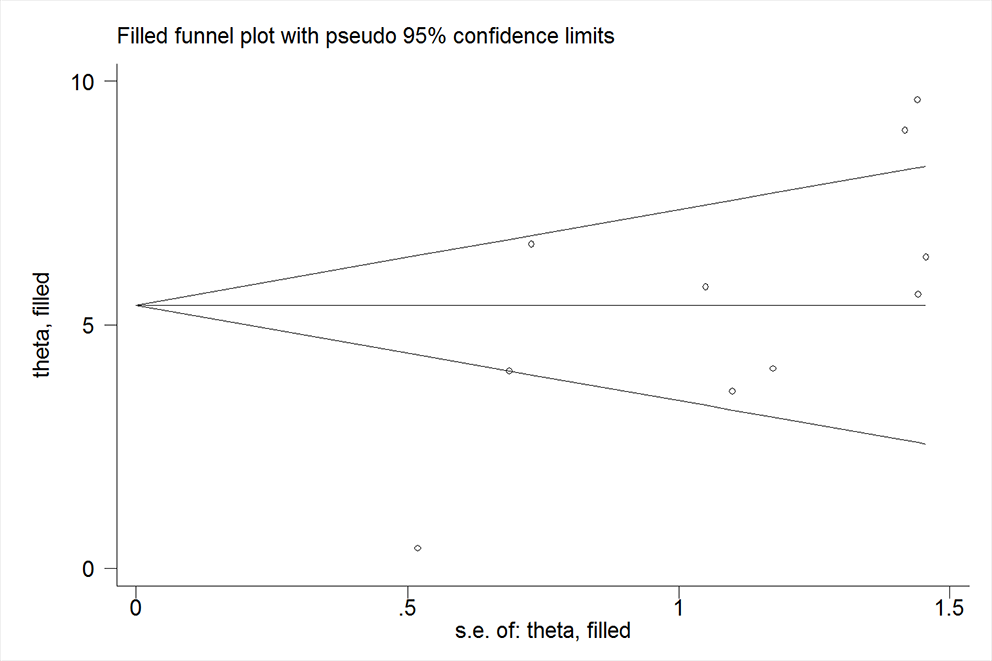

Supplement: Figure S1 — Filled funnel plot of any PEP using trim-and-fill method. [file Image_1.TIF]
